# Condition-specific surveillance in health care-associated urinary tract infections as a strategy to improve empirical antibiotic treatment: an epidemiological modelling study

**DOI:** 10.1007/s00345-019-02963-9

**Published:** 2019-09-25

**Authors:** Zafer Tandogdu, Bela Koves, Tommaso Cai, Mete Cek, Peter Tenke, Kurt Naber, Florian Wagenlehner, Truls Erik Bjerklund Johansen

**Affiliations:** 1 Institute of Clinical Medicine, University of Oslo, Oslo, Norway; 2 Department of Urology, University College London Hospitals, London, UK; 3 Department of Urology, South-Pest Teaching Hospital, Budapest, Hungary; 4 Department of Urology, Santa Chiara Regional Hospital, Trento, Italy; 5 Department of Urology, Trakya Medical School, Edirne, Turkey; 6 Technical University of Munich, Munich, Germany; 7 Department of Urology, Pediatric Urology and Andrology, Justus Liebig University Giessen, Giessen, Germany; 8 Department of Urology, Oslo University Hospital, Oslo, Norway; 9 Institute of Clinical Medicine, University of Aarhus, Aarhus, Denmark; 10 Division of Surgery and Interventional Science, University College London, London, UK

**Keywords:** Antibiotic stewardship, Health care-associated UTI, Condition-specific surveillance

## Abstract

**Background:**

Health care-associated urinary tract infection (HAUTI) consists of unique conditions (cystitis, pyelonephritis and urosepsis). These conditions could have different pathogen diversity and antibiotic resistance impacting on the empirical antibiotic choices. The aim of this study is to compare the estimated chances of coverage of empirical antibiotics between conditions (cystitis, pyelonephritis and urosepsis) in urology departments from Europe.

**Methods:**

A mathematical modelling based on antibiotic susceptibility data from a point prevalence study was carried. Data were obtained for HAUTI patients from multiple urology departments in Europe from 2006 to 2017. The primary outcome of the study is the Bayesian weighted incidence syndromic antibiogram (WISCA) and Bayesian factor. Bayesian WISCA is the estimated chance of an antibiotic to cover the causative pathogens when used for first-line empirical treatment. Bayesian factor is used to compare if HAUTI conditions did or did not impact on empirical antibiotic choices.

**Results:**

Bayesian WISCA of antibiotics in European urology departments from 2006 to 2017 ranged between 0.07 (cystitis, 2006, Amoxicillin) to 0.89 (pyelonephritis, 2009, Imipenem). Bayesian WISCA estimates were lowest in urosepsis. Clinical infective conditions had an impact on the Bayesian WISCA estimates (Bayesian factor > 3 in 81% of studied antibiotics). The main limitation of the study is the lack of local data.

**Conclusions:**

Our estimates illustrate that antibiotic choices can be different between HAUTI conditions. Findings can improve empirical antibiotic selection towards a personalized approach but should be validated in local surveillance studies.

**Electronic supplementary material:**

The online version of this article (10.1007/s00345-019-02963-9) contains supplementary material, which is available to authorized users.

## Introduction

Health care-associated urinary tract infection (HAUTI) is a major concern with a prevalence of 7.7% (4.6–17.3%) in urology departments, which is higher compared to other departments (0.5–1.7%) [1–4]. HAUTIs consist of three main clinical conditions that include cystitis, pyelonephritis and urosepsis. In severe HAUTIs, timely administration of antibiotics is crucial with delays increasing the risk of morbidity and mortality [5–9]. Lack of rapid microbiological diagnostics means that first-line antibiotic selection is carried out in the absence of individual patient-specific antibiograms (empirical antibiotics). Empirical antibiotic selection process requires judicious assessment of prior information such as the patient-specific conditions and local antibiotic resistance information [9]. Otherwise, selected empirical antibiotics for infections can be in discordance with the causative pathogen in up to 50% of cases leading to inappropriate antibiotic usage [10, 11].

Selection of empirical antibiotics can be improved with infection surveillance data [12]. As part of routine surveillance studies, HAUTIs are reported as an individual clinical diagnostic entity [12, 13]. This can be problematic because HAUTI is an umbrella term covering different infectious conditions of the urinary tract (cystitis, pyelonephritis and urosepsis), and, therefore, a condition-specific discrepancy of antibiotic resistance (AMR) can be expected [1]. In our previous work, AMR rates were higher in urosepsis for specific antibiotics compared to pyelonephritis and cystitis [1]. Additionally, pathogen diversity between conditions can also be different. The combination of difference in pathogen diversity with AMR can impact the empirical choices between conditions.

Comparison of empirical choices between conditions can be achieved using a composite index called weighted incidence syndromic combination antibiogram (WISCA) [14, 15]. WISCA measures the chances of coverage of the possible causative pathogens for each antibiotic choice (single or combination). This allows a direct comparison between different HAUTI conditions. Pathogen and AMR data derived from sequential surveillance studies include analytical challenges that can be overcome using Bayesian methods [16, 17]. In a recent study, we demonstrated that the Bayesian WISCA alongside probabilistic methods were useful in determining the impact of infection control policies in HAUTIs [15].

In the present study, we hypothesized that due to discrepancies of pathogens and AMR between HAUTI conditions, the chances of coverage of empirical antibiotic choices (WISCA) will be different. The aim of this study is to compare the estimated chances of coverage of empirical antibiotics between conditions (cystitis, pyelonephritis and urosepsis) in urology departments from Europe based on large sequential surveillance data. If the hypothesis is supported with strong evidence amongst a range of antibiotics, it will create an opportunity to improve empirical antibiotic selection for future patients with HAUTIs. In other words, an individualized approach for empirical antibiotics will be justified.

## Materials and methods

### Data collection

Data were obtained from the Global Prevalence of Infections in Urology (GPIU) point prevalence study [Clinicaltrials.gov iD NCT03665467] [18]. The study was conducted annually (same days of November) in urology departments with a web-based platform [18]. Ethical approval for the study was at the department’s discretion for the study years 2003–2006. In 2007, a central ethical approval from Giessen University ethical committee in Germany was obtained which did not require informed consent from participants. The study is sponsored by the European Association of Urology Research Foundation (EAU-RF).

On the study day, all patients in urology wards are screened for HAUTI and surgical site infections (SSI) as defined by the Center for Disease Control and Prevention (CDC) criteria (Supplement I) [5]. Clinical information including culture specimens is collected from patients with an episode of HAUTI or SSI.

### Outcomes

Outcomes of the study are:

- Prevalence of HAUTIs, pathogens and AMR of pathogens,
- Diversity of causative pathogens within conditions of HAUTIs using the Shannon diversity index [19],
- Dissimilarity of causative pathogens between conditions of HAUTIs using the Bray–Curtis index [19],
- Chances of an antibiotic to provide coverage for a condition using the Bayesian WISCA [15, 17],
- Differences of Bayesian WISCA estimates between conditions using the Bayesian factor [20].

Geographical variability was accounted for by grouping countries based on the 2017 European Center for Disease Control (ECDC) AMR prevalence data [21]. Countries with a pooled AMR rate below 5% were categorized as low and those with a rate above 5% as high resistance (Supplement II).

### Data analysis

Data analysis was carried out in R version 3.5.1.

#### Pathogens

Pathogen diversity within clinical conditions was illustrated with the Shannon diversity index [19]. Pathogen dissimilarity between clinical conditions was evaluated with the Bray–Curtis index [19]. The index ranges between 0 (two infection sites have identical pathogens) to 1 (two infection sites have different pathogens). It was assumed that each infective condition is independent of the other.

#### AMR

AMR rates were calculated for 11 single and 10 combination choices as recommended by international guidelines for HAUTIs (Supplement III) [22]. Antibiotic susceptibility tests were performed by participating institutions using routine methods. For combination antibiotic choices, possible synergistic interactions and common resistance mechanisms were not considered. Antibiotics are presented with relevant international abbreviations (Supplement III). Data were reported as sensitive, intermediate or resistant and for analysis, all intermediate groups were assumed resistant.

#### Bayesian WISCA

Bayesian WISCA represents the probability of an empirical antibiotic choice to cover the causative pathogens. Calculation involves two stages and two variables: (i) probability of etiological pathogens; and (ii) probability of each etiological pathogen to be susceptible towards the antibiotic. These probabilities are obtained from the GPIU prevalence data by applying the relevant functions (Supplement IV). After obtaining the two probabilities, they are multiplied and added.

The Bayesian approach accounts for prior information of pathogens and their resistance to antibiotics [17]. Hierarchical modelling techniques were used on the GPIU data from the preceding 2 years of each studied year to calculate the informative priors. This is then combined with the raw data to obtain the posterior distributions (final probabilities) for each studied year.

#### Comparison of Bayesian WISCA estimates between conditions

The Bayesian factor was used to determine if the chances of coverage of an antibiotic will vary between HAUTI conditions. This was achieved by obtaining the likelihood of two competing hypothesis and calculating their ratio [20]. The two competing plausible hypotheses in this study were:

- *H*_0_: Dissimilarity of pathogens and their respective AMR between conditions does not impact the chances of coverage of empirical antibiotic.
- *H*_*1*_: Dissimilarity of pathogens and their respective AMR between conditions impacts the chances of coverage of empirical antibiotic.

The Bayes factor quantifies the strength of evidence to support or refute the concept that HAUTI conditions impact the Bayesian WISCA. Analysis of variance (ANOVA) models were developed to account for geographic and time variability of Bayesian WISCA estimates. The likelihood probabilities for each hypothesis were calculated through the ANOVA models. Conceptually the models were as follows:

- *Model 1* (*M1)*: Bayesian WISCA of antibiotic ~ study year (2006–2017) + regions of resistance (high vs low).
- *Model 2* (*M2*): Bayesian WISCA of antibiotic ~ study year (2006–2017) + regions of resistance (high vs low) + HAUTI conditions (cystitis, pyelonephritis and urosepsis).

Likelihood of model 2 was divided with the likelihood of model 1 to compute the Bayes factor (BF_M2vsM1_). Interpretation of the Bayes factor was conducted using the classification proposed by Jeffreys [23] (Table 1).

**Table 1:** Interpretation of Bayes factor to accept or refute the concept that HAUTI conditions can impact the Bayesian WISCA estimates

BF_M2vsM1_	Evidence to support HAUTI conditions impact on Bayesian WISCA
1–3	Anecdotal
3–10	Substantial
10–30	Strong
30–100	Very strong
> 100	Decisive

## Results

During the 12-year surveillance, 18,447 patients were screened in Europe and HAUTI was documented in 9.6% (*n* 1767) of them, which 7.6% (*n* 1398) were confirmed with culture tests. Summary of patient acquisition according to STROBE guidance is provided in Supplement V.

Mean age was 63.4 (SD 16.6) and mean Charlson comorbidity score was 2.4 (SD 2.7) (Supplement VI). Frequency of clinical conditions was: 38.0% cystitis, 31.6% pyelonephritis and 30.3% urosepsis. Mean Charlson score was similar in clinical conditions (*p* = 0.09). A Charlson score above 1 was measured in 62.2% of patients with urosepsis (significantly higher than other conditions *p* = 0.000). Nephrostomies, ureteral stents and ureteric stones were more common in pyelonephritis and urosepsis compared to cystitis (*p* = 0.000, Supplement VI).

### Pathogens in HAUTIs

**Fig. 1:**
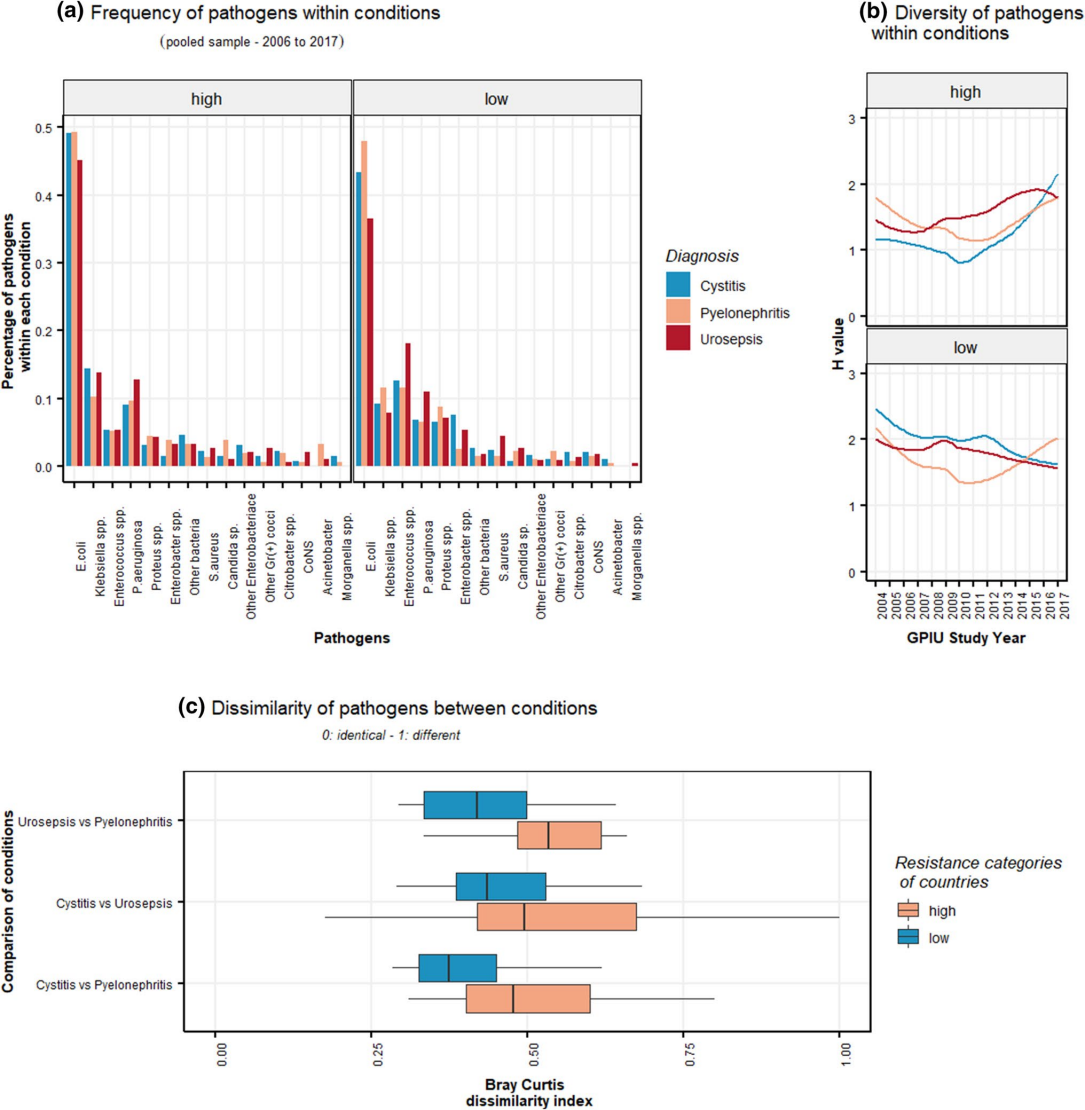
Pathogen frequency (**a**) and diversity (**b**) within conditions and dissimilarity between conditions (**c**) stratified according to high- and low-resistant countries. Ranking of pathogens are similar amongst conditions. The dissimilarity between conditions is apparent when studied with Bray–Curtis index. The Bray–Curtis index was analysed for each year but to illustrate that its range was not reported in chronological order

The frequency of pathogens ranked in HAUTIs overall was: *E. coli *> *Klebsiella* spp. > *P. aeruginosa* (Fig. 1a). Ranking of pathogens was similar amongst conditions, European regions, Charlson comorbidity groups and study periods (logistic-regression analysis *p* > 0.05). Median Shannon diversity index over the study periods was 1.2 and 2.1 for cystitis, 1.6 and 1.8 for pyelonephritis, and 1.4 and 1.7 in low- and high-resistant countries, respectively (Fig. 1b). Median Bray–Curtis dissimilarity index over the years was 0.53 and 0.42 for pyelonephritis vs urosepsis, 0.47 and 0.37 for pyelonephritis vs cystitis, and 0.49–0.43 for cystitis vs urosepsis in low- and high-resistant countries, respectively (Fig. 1c).

### AMR

AMR rates in the pooled sample of pathogens and years (2003–2017) ranged between 8.2% (imipenem) and 62.6% (amoxicillin) for single antibiotic choices and 16.3% [piperacillin/tazobactam (TZP) + GEN] to 32.3% [ciprofloxacin (CIP) + trimethoprim/sulfamethoxazole (TMP)] combination choices (Supplement VII). For *E. coli* (n: 615) in the pooled sample of years, the AMR rates ranged between 3.1% [Imipenem (IMP)] to 58.1% (AMX) for single antibiotic choices and 10.8% (TZP + GEN) to 28.9% (CIP + TMP) for combination choices (Supplement VII).

### Bayesian WISCA estimates

**Fig. 2:**
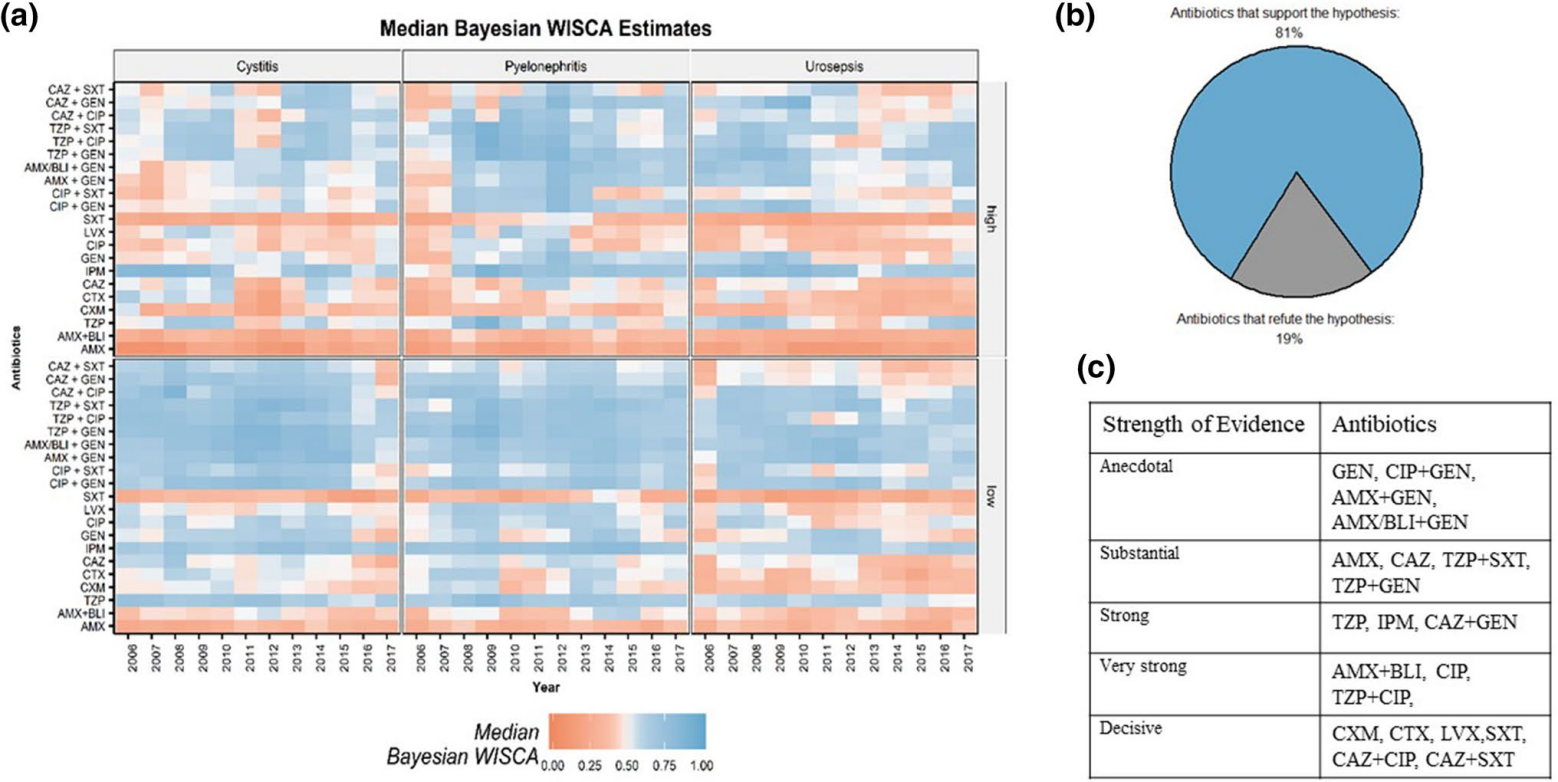
Median Bayesian WISCA estimates of antibiotic choices for each year and HAUTI subgroup (cystitis, pyelonephritis and urosepsis) stratified according to lower vs higher frequency-resistant countries (stratification was carried out based on the ECDC reported pooled *E. coli* resistance frequencies. The 5% threshold was used)

Pooled data—all HAUTIs: Bayesian WISCA estimates ranged between 0.09 (IQR 0.07–0.12, AMX, 2006, high-resistant countries) and 0.86 (IQR 0.83–0.88, TZP + SXT, 2012, low-resistant countries). Estimated median Bayesian WISCA per condition is illustrated in Fig. 2a.

*Cystitis*: estimates for single agents ranged between 0.07 (IQR 0.03–0.17, AMX, 2006, high-resistant countries) and 0.84 (IQR 0.80–0.87, IPM, 2007, high-resistant countries).

*Pyelonephritis*: single agent median estimates were between 0.14 (IQR 0.10–0.19, AMX, 2006, high-resistant countries) and 0.89 (IQR 0.85–0.91, IPM, 2009, low-resistant countries). Combination choice median estimates were lowest at 0.36 [IQR 0.30–0.42, Ceftazidime (CAZ) +GEN, 2007, high-resistant countries] and highest at 0.89 (IQR 0.86–0.92, TZP + CIP, 2009, high-resistant countries).

*Urosepsis*: single agent median estimates were between 0.11 (IQR 0.07–0.15, AMX, 2010, high-resistant countries) and 0.83 (IQR 0.79–0.86, IPM, 2010, low-resistant countries). Combination choice median estimates were lowest at 0.29 (IQR 0.83–0.89, CAZ + SXT, 2006, low-resistant countries) and highest at 0.86 (IQR 0.86–0.92, CAZ + GEN, 2010, high-resistant countries).

### Comparison of Bayesian WISCA estimates between conditions

A relationship between the clinical conditions and the chances of coverage of antibiotics was rejected in 19% of the antibiotics [GEN and its combinations with CIP, AMX and AMX/Beta lactamase inhibitor (BLI)] (Fig. 2b). For the remaining 81% of the studied antibiotics, the relationship with the Bayesian WISCA was confirmed. Level of evidence for each antibiotic is illustrated in Fig. 2c.

## Discussion

In this study, we hypothesized that distinct patterns of pathogen diversity and AMR between conditions would lead to differences in empirical antibiotic choices. Chances of coverage of 21 antibiotic choices were studied and condition-specific differences were supported in 81% of them. We achieved this by obtaining the Bayesian WISCA of antibiotics per year and compared each antibiotic choice between conditions using the Bayesian factor. We also accounted for geographical variation by separating the European countries into high and low resistance areas using the ECDC surveillance data.

Frequencies of pathogens both in our previous and current (Fig. 1a) study were similar between the conditions [1]. Due to the large variety and heterogeneity of causative pathogens in HAUTI conditions, frequencies can be inadequate for comparison. We, therefore, employed diversity indices to quantify the pathogen diversity (Shannon index) within conditions and dissimilarity (Bray–Curtis index) between conditions [19]. The Bray–Curtis index revealed a dissimilarity of pathogen diversity between conditions (Fig. 1c). This dissimilarity could reflect the different responses elicited by bacteria and/or host leading to different clinical manifestations. Studies investigating the genetic or phylogenetic classifications alongside the host response can improve our understanding of the impact of pathogen diversity amongst conditions. This can help explore the host–pathogen relationship and individualize managements.

In our analysis, the AMR frequency of *E. coli* showed differences between conditions in 6 (28%) out of 21 studied antibiotics. These findings were similar to our previous published results in 2016 and illustrate that AMR can be different for pathogens between conditions but it does not explain the coverage of an empirical antibiotic [1, 15]. We, therefore, used the Bayesian WISCA that estimated a difference in 81% of studied antibiotics. There was decisive evidence to support the difference between conditions for the following single agents: cefuroxime, cefotaxime, levofloxacin and trimethoprim + sulfamethoxazole. Overall, a lower Bayesian WISCA for urosepsis was noted for all antibiotics compared to other conditions. The only antibiotic that did not follow this trend was gentamicin. The estimates obtained from this study support the concept that chances of coverage of antibiotics are different between conditions. It is likely that condition-specific surveillance can improve antibiotic stewardship and contribute as a strategy to tackle AMR in HAUTIs. A study by Bielicki et al. illustrated that continental level surveillance information would not be accurate to guide local empirical antibiotic selection [17]. Therefore, antibiotic stewardship programs for HAUTIs conditions that utilize surveillance data should test these findings at a local level. A recent study from The Netherlands reported WISCA values of urosepsis and HAUTIs, for which they attempted to provide recommendations for empirical antibiotic treatments [24]. However, due to lack of clinical information and retrospective nature, the recommendations would still be inaccurate. Therefore, we also suggest that WISCA estimates should be used for empirical recommendations if only local protocol-driven clinical data derived through validated definitions for clinical conditions of HAUTIs are available. We expect that HAUTI condition-specific Bayesian WISCA estimates can help in improving empirical antibiotic treatment of HAUTI conditions. Thus, the risk of morbidity and mortality decreases.

The strength of the study is based on two main factors. First, the GPIU surveillance to our knowledge is the only study that has a sequential measure of HAUTIs in urology departments using validated definitions. This has generated data comparable over time. Second, analysis of the data utilizing the Bayesian WISCA approach has allowed us to unmask previously unknown findings. The WISCA measure has been used before [14, 24] and inclusion of the Bayesian approach has only been of note in two other studies [15, 17]. In our recent study, we established the methodology for the Bayesian approach in WISCA estimates [15]. Accounting for the prior information improved the estimates. In addition, the Bayesian factor was used to test the hypothesis that chances of coverage of an antibiotic will differ amongst conditions. This was compared against the competing hypothesis of no such relation and allowed us to judge the strength of the evidence obtained from the GPIU study to support or refute the hypothesis.

Although this study has demonstrated that condition-specific surveillance information in HAUTIs can improve the information obtained for the empirical antibiotic selection, there are several limitations. First, the data are derived from an annual surveillance study at a continental level and participation from each country was varied. Hence, the accuracy of the results is questionable but previous studies have identified that the inaccuracy is more likely to be gauged towards an underestimate of AMR [25, 26]. Second, we used a 2-year prior information to calculate the Bayesian WISCA and the value of a longer duration for prior information should be further explored.

## Conclusions

Our estimates illustrate that antibiotic choices can be different between HAUTI conditions. The Bayesian WISCA approach was instrumental in highlighting these differences. The findings can improve empirical antibiotic selection towards a personalized approach but need to be validated in local surveillance studies.

## Electronic supplementary material

Below is the link to the electronic supplementary material.

Supplementary material 1 (DOCX 2385 kb)
