# Supplementary material for: Condition-specific surveillance in health care-associated urinary tract infections as a strategy to improve empirical antibiotic treatment: an epidemiological modelling study
Source: World J Urol. 2019 Sep 25;38(1):27–34. doi: 10.1007/s00345-019-02963-9 (PMC6954147; doi:10.1007/s00345-019-02963-9)

**Supplement**

Contents

[1. Definitions of Infections used in the Global Prevalence of Infections in Urology Study 2](#_Toc9630769)

[2. Stratification of countries as low and high resistance 4](#_Toc9630770)

[3. Antibiotic choices evaluated 5](#_Toc9630771)

[4. Obtaining probabilities and calculation of Bayesian WISCA of an antibiotic 6](#_Toc9630772)

[4.1. Hierarchical modelling to obtain prior probability distributions: 6](#_Toc9630773)

[4.2. Obtaining Probability of Etiological pathogens and antibiotic susceptibility: 6](#_Toc9630774)

[4.3. Definition of Bayesian WISCA on an antibiotic: 6](#_Toc9630775)

[5. GPIU Study Patient Case Disposition 7](#_Toc9630776)

[6. Demographics of patients with HAUTIs in Europe 8](#_Toc9630777)

[7. AMR of HAUTIs in Europe (2003-2017) 10](#_Toc9630778)

[7.1. AMR Rates of HAUTI Subgroups in Europe – Pooled Sample 10](#_Toc9630779)

[7.2. Time trends of AMR rates in Europe within the pooled sample of all pathogens 13](#_Toc9630780)

[*7.3.* Time trends of AMR rates in Europe for *E.coli* 15](#_Toc9630781)

[8. Charlson Comorbidity Score and Bayesian WISCA Values 17](#_Toc9630782)

# Definitions of Infections used in the Global Prevalence of Infections in Urology Study

**Urinary Tract Infection:** Urinary tract infection includes symptomatic urinary tract infection, asymptomatic bacteriuria, and other infections of the urinary tract.

**Symptomatic urinary tract infection** must meet one of the following criteria:

1. One of the following: fever (>38°C), urgency, frequency, dysuria, or suprapubic tenderness AND a urine culture* of >= 10^5^ colonies/ml urine with no more than two species of organisms
2. Two of the following: fever (>38°C), urgency, frequency, dysuria, or suprapubic tenderness AND any of the following:

- Dipstick test positive for leukocyte esterase and/or nitrate.
- Pyuria (>=10 white blood cells (WBC)/ml or >=3 WBC/high-power field of unspun urine).
- Organisms seen on Gram stain of unspun urine
- Two urine cultures with repeated isolation of the same uropathogen^+^ with 10^2^ colonies/ml urine in nonvoided specimens
- Urine culture with <=10^5^ colonies/ml urine of single uropathogen in patient being treated with appropriate antimicrobial therapy.
- Physician's diagnosis.
- Physician institutes appropriate antimicrobial therapy.

1. Patient <=12 months of age has one of the following: fever (>38°C), hypothermia (<37°C), apnea, bradycardia, dysuria, lethargy, or vomiting AND urine culture of 10^5^ colonies/ml urine with no more than two species of organisms.
2. Patient <=12 months of age has one of the following: fever (>38°C), hypothermia (>37°C), apnea, bradycardia, dysuria, lethargy, or vomiting AND any of the following:

- Dipstick test positive for leukocyte esterase and/or nitrate.
- Pyuria.
- Organisms seen on Gram stain of unspun urine.
- Two urine cultures with repeated isolation of same uropathogen with >=10^2^ organisms/ml urine in nonvoided specimens.
- Urine culture with <=10^5^ colonies/ml urine of a single uropathogen in patient being treated with appropriate antimicrobial therapy.
- Physician's diagnosis.
- Physician institutes appropriate antimicrobial therapy.

**Asymptomatic bacteriuria** must meet either of the following criteria:

1. An indwelling urinary catheter is present within 7 days before urine is cultured AND patient has no fever (>38°C), urgency, frequency, dysuria or suprapubic tenderness AND has urine culture of 10^5^ organisms/ml urine with no more than two species of organisms.
2. No indwelling urinary catheter is present within 7 days before the first of two urine cultures with >=10^5^ organisms/ml urine of the same organism with no more than two species of organisms, AND patient has no fever(>38°C), urgency, frequency, dysuria, or suprapubic tenderness.

**Other infections of the urinary tract** (kidney, ureter, bladder, urethra, or tissues surrounding the retroperitoneal or perinephric spaces) must meet one of the following criteria:

1. Organism isolated from culture of fluid (other than urine) or tissue from affected site
2. An abscess or other evidence of infection seen on direct examination, during surgery, or by histopathologic examination
3. Two of the following: fever(>38°C), localized pain, or tenderness at involved site AND any of the following:

- Purulent drainage from affected site.
- Organism isolated from blood culture.
- Radiographic evidence of infection*.
- Physican's diagnosis.
- Physican institutes appropriate antimicrobial therapy.

1. Patient <=12 months of age has one of the followin: fever (>38°C), hypothermia (<37°C), apnea, bradycardia, lethargy, or vomiting AND any of the following:

- Purulent drainage from affected site.
- Organism isolated from blood culture.
- Radiographic evidence of infection.
- Physician's diagnosis.
- Physician instituted appropriate therapy.

*For urine specimens to be of value in determining whether a nosocomial infection exists, they must be obtained aseptically using an appropriate technique, such as clean catch collection, bladder catheterization, or suprapubic aspiration.

+Gram-negative bacteria of Staphylococcus saprophyticus.

**Urosepsis definitions and classification used in the GPIU study*:**

| Disorder | Definition |
| --- | --- |
| Urosepsis (simple) | Activation of a systemic inflammatory response syndrome (SIRS) due to a urinary tract infection (UTI). This systemic response is manifested by two or more of the following conditions:  Temperature > 38°C or < 36°C.  Heart rate > 90 beats min.  Respiratory rate > 20 breaths/min or PaCO2 < 32mmHg (< 4.3kPa).  WBC > 12,000 cells/mm^3^ or < 4,000 cells/mm^3^ or ≥ 10% immature (band) forms. |
| Severe Urosepsis | Urosepsis associated with organ dysfunction, hypoperfusion or hypotension.  Hypoperfusion and perfusion abnormalities may include but are not limited to lactic acidosis, oliguria or an acute alteration of mental status. |
| Uroseptic Shock | Urosepsis with hypotension despite adequate fluid resuscitation along with the presence of perfusion abnormalities that may include, but are not limited to lactic acidosis, oliguria, or an acute alteration in mental status.  Patients who are on inotropic or vasopressor agents may not be hypotensive at the time that perfusion abnormalities are measured. |

*New sepsis definitions (Sepsis-3) have not been used as part of this study (Singer M, Deutschman CS, Seymour CW, Shankar-Hari M, et al. The Third International Consensus Definitions for Sepsis and Septic Shock (Sepsis-3).JAMA. 2016 Feb 23;315(8):801-10. doi: 10.1001/jama.2016287)

# Stratification of countries as low and high resistance

Countries were classified as low or high resistance areas based on the European Center for Disease Control (ECDC) 2018 AMR surveillance report. The ECDC report provides AMR rates of pathogens towards a range of antibiotics. *E.coli* resistance towards third generation cephalosporins, fluoroquinolones and aminoglycosides was used to categorize countries as low or high resistant areas. A resistance rate of 5% was used as a threshold. The countries data was obtained from in the GPIU study and their respective categories is summarized below.

| Low resistance | High resistance |
| --- | --- |
| Austria | Bulgaria |
| Belgium | Croatia |
| Denmark | Czech Republic |
| Estonia | Greece |
| Finland | Hungary |
| France | Italy |
| Germany | Poland |
| Netherlands | Portugal |
| Norway | Romania |
| Sweden | Slovakia |
| UK | Spain |

Countries with no ECDC data reported:

1. Albania
2. Bosnia and Herzegovina
3. Macedonia (FYROM)
4. Serbia
5. Switzerland
6. Turkey

Countries without ECDC data were categorized based on previous GPIU study publications and data. Apart from Switzerland all other five countries were categorized as high resistant areas.

# Antibiotic choices evaluated

The antibiotic choices evaluated were obtained from the list of recommendations of the European Association of Urology guidelines published in 2018. Combination classes were determined by using antibiotic classes that did not have common resistance mechanisms.

s-Table 1, Antibiotic choices evaluated for empirical treatment of HAUTIs in urology.

| Single antibiotic choices | Combination antibiotic choices |
| --- | --- |
| Amoxicillin | Amoxicillin+Gentamicin |
| Aminopenicillin/Beta lactamase inhibitor (Amp/BLI) | Amp/BLI+Gentamicin |
| Piperacillin/Tazobactam(TZB) | Ceftazidime+Gentamicine |
| Cefuroxime | Ceftazidime+ Ciprofloxacin |
| Cefotaxime | Ceftazidime+ Trimethroprim /Sulfametaxazole |
| Ceftazidime | Piperacillin/Tzb + Gentamicin |
| Ciprofloxacin | Piperacillin/Tzb+ Ciprofloxacin |
| Levofloxacin | Piperacillin/Tzb+ Trimethroprim /Sulfametaxazole |
| Trimethroprim + Sulfametaxazole | Ciprofloxacin+ Gentamicin |
| Gentamicin | Ciprofloxacin+ Trimethroprim /Sulfametaxazole |
| Imipenem |  |

List of antibiotic abbreviations:

- Amoxicillin: AMX
- Aminopenicillin/Beta lactamase inhibitor: AMX+BLI
- Piperacillin/Tazobactam: TZP
- Cefuroxime : CXM
- Cefotaxime : CTX
- Ceftazidime : CAZ
- Ciprofloxacin : CIP
- Levofloxacin : LVX
- Trimethroprim + Sulfametaxazole : TMP+SMX
- Gentamicin : GEN
- Imipenem : IPM

# Obtaining probabilities and calculation of Bayesian WISCA of an antibiotic

## Hierarchical modelling to obtain prior probability distributions:

The prior probability distribution was obtained from the GPIU data of the preceding two years of studied year [21]. Hierarchical modelling in the Bayesian framework can be used when data or its sources is organized in groups. In this context, the two study years before the year of interest are assessed as distinct groups that will be used to update our final probability estimate. The prior information ($\theta$) used to construct the conjugate posterior ($\omega$) will be assigned a prior distribution with unknown hyperparameters ($\varphi$). This hyperparameter will be assigned a distribution, which will be used to determine and update the conjugate posterior probability.

The Bioconductor package in “R” was used to obtain the hyperparameters (prior) for the pathogen distribution and the sensitivity [22].

## Obtaining Probability of Etiological pathogens and antibiotic susceptibility:

The probability of each pathogen to be causative of a HAUTI is obtained from the GPIU prevalence data. The probabilities of etiological pathogens are sampled through the Dirichlet distribution (p_1(t)_,…,p_n_), where p_j_ is the probability of pathogen (j). Informative prior distribution (p_1(t-1 & t-2)_,…,p_n(t-1 & t-2)_) is obtained using the hierarchical modelling explained above.

Subsequently the conjugate posterior Dirichlet probability distribution (p_1(t-1 & t-2)_+ p_1(t)_,…,p_n(t-1 & t-2)_+p_n(t)_) was obtained.

Antibiotic susceptibility profile data in the GPIU was gathered as: resistant (r), intermediate and sensitive (s). Pathogen susceptibility profile was determined for 11 single agent antibiotics and 10 combination options, which are appropriate for use in UTIs (s-table-1). Each of these antibiotic options has a known antibacterial spectrum with a certain in-vitro and in–vivo activity. This list was obtained from European Committee on Antimicrobial Testing (EUCAST) consensus [30]. For combination options, pathogens were considered sensitive if they were sensitive to at least one of the antibiotics. Possible synergistic, additional or antagonistic action of antibiotic combinations has not been taken into account.

Susceptibility profile (sensitive or not) was assumed to have a binomial distribution and the appropriate Beta-distribution was applied [20]. Prior distribution was assumed to be informative and was obtained as explained in step 1 (sensitive _(t-1 & t-2)_, resistant _(t-1 & t-2)_). Subsequently, the conjugate posterior distribution (sensitive _(t-1 & t-2)_+ sensitive _(t)_, resistant _(t-1 & t-2)_+ resistant _(t)_) was obtained .

For each antibiotic this was obtained for pathogens part of their susceptibility profile.

## Definition of Bayesian WISCA on an antibiotic:

For an infection there would be (m) number of causative pathogens. Running the Dirichlet distribution provides p_j_ probability of having pathogen j.

For this infection there are (n) number of antibiotics that can be used. Running the Beta distribution provides q_ij_ probability of the pathogen j being sensitive to Antibiotic i. Two inherent conditions apply for q_ij_ probability to be 0. First; the pathogen is intrinsically resistant to the antibiotic. Second, the pathogen is not part of the antibiotic susceptibility profile. Followed the total sampled probability of being sensitive to Antibiotic(A*i*) is

$$WISCA\left( A_{i} \right)= \sum_{j=1}^{m} q_{ij}p_{j}$$

The parameter i runs over the values 1 to n.

# GPIU Study Patient Case Disposition

s-Figure 1, Patient case disposition from the GPIU for the current study.
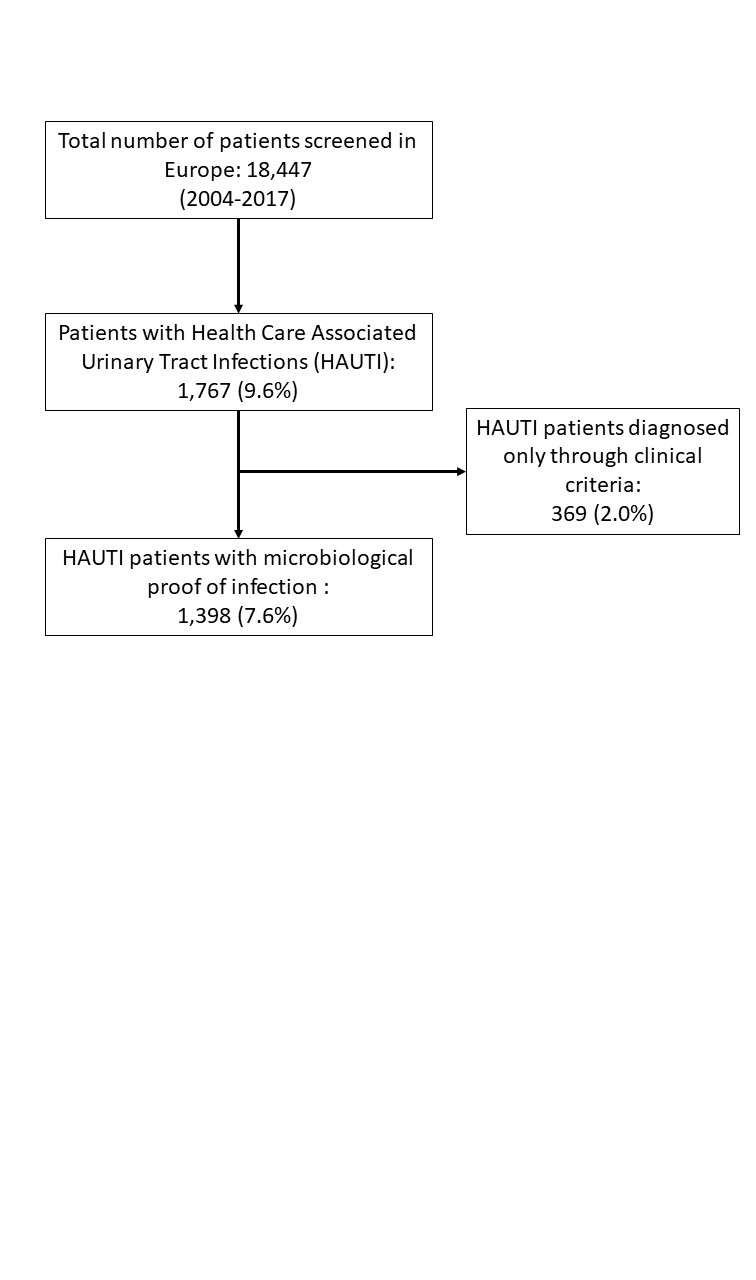


s-Figure 2, Number of cases surveyed, prevalence of HAUTIs with microbiological proof of infection only and prevalence of HAUTIs including cases diagnosed with clinical criteria only in Europe from 2004 to 2017.


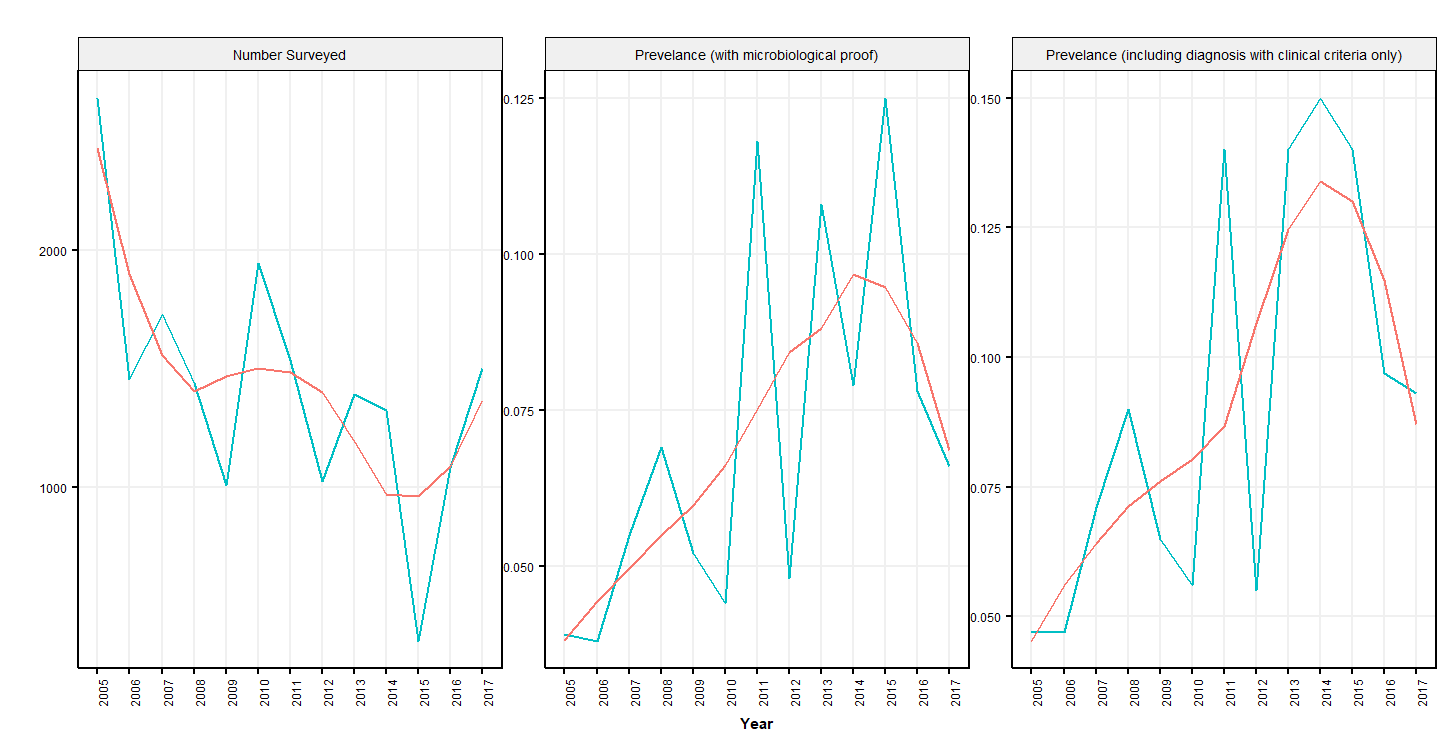


# Demographics of patients with HAUTIs in Europe

s-Table 2, Summary of risk factors in HAUTI conditions from the pooled GPIU data (2003-2017)

|  | | | | Cystitis | Pyelonephritis | Urosepsis | Total |
| --- | --- | --- | --- | --- | --- | --- | --- |
|  |  |  |  | 38.0%  (527) | 31.6%  (438) | 30.3%  (421) | 1386 |
| Age |  |  | |  |  |  |  |
| Charlson comorbidity score | Overall score | Mean (SD) | | 2.3 (2.7) | 2.2 (2.9) | 2.6 (2.6) | 2.4(2.7) |
|  |  | p value (ANOVA) | | 0.09 | | |  |
|  | Stratified | >1 | % (n) | 51.4%  (271) | 46.6%  (204) | 62.2%  (262) | 53.2%  (737) |
|  |  |  | p | - | 0.1 | 0.000 | Goodnes-to-fit=0.000 |
|  |  |  | OR (95% CI) | - | - | 1.5 (1.2-2.0) |  |
|  |  | >2 | % (n) | 35.7%  (188) | 33.1%  (145) | 39.4%  (166) | 36.0%  (499) |
|  |  |  | p | - | 0.4 | 0.2 | NS |
| Catheters | Patients with a catheter | % (N) | | 62.0%  (327) | 54.3%  (238) | 61.0%  (257) | 59.3%  (822) |
|  |  | p value¬ | | - | 0.01 | 0.7 | Goodnes-to-fit=0.03 |
|  |  | OR (95% CI) | | - | 0.7 [0.5-0.9] | - |  |
|  | Number of catheters in patients with a catheter | 1  % (N) | | 56.5%  (298) | 42.2%  (185) | 45.6%  (192) | 48.7%  (675) |
|  |  | >1  % (N) | | 5.5%  (29) | 12.1%  (53) | 15.4%  (65) | 10.6%  (147) |
|  |  | p value¬ | | - | 0.000 | 0.000 |  |
|  |  |  | | - |  |  |  |
|  | **Nephrostomy** | (+)  % (N) | | 2.5%  (n:12) | 22.2%  (n:86) | 17.7%  (n:67) | 13.3% (n:165) |
|  |  | unknown  % (N) | | 10.6%  (56) | 11.6%  (51) | 10.2%  (43) | 10.8%  (150) |
|  |  | p value** | | - | 0.00 | 0.00 | Goodnes-to-fit=0.000 |
|  |  | OR (95% CI) | | - | 7.8[3.8-16.5] | 7.2[3.5-15.1] |  |
|  | **Urethral** | (+)  % (N) | | 53.1%  (264) | 20.8%  (80) | 33.5%  (262) | 37.3%  (476) |
|  |  | unknown  % (N) | | 5.7%  (30) | 12.5%  (55) | 6.4%  (27) | 8.3%  (112) |
|  |  | p value** | | - | 0.000 | 0.000 | Goodnes-to-fit=0.000 |
|  |  | OR (95% CI) | | - | 0.2[0.1-0.3] | 0.6[0.3-0.9] |  |
|  | Suprapubic | (+)  % (N) | | 7.6%  (36) | 5.0%  (19) | 8.0%  (30) | 6.9%  (85) |
|  |  | unknown  % (N) | | 10.0%  (53) | 13.9%  (19) | 10.9%  (46) | 11.5%  (160) |
|  |  | p value** | | NS | | | Goodnes-to-fit=0.13 |
|  | **Ureteral stent** | (+)  % (N) | | 7.8%  (37) | 25.3%  (99) | 20.4%  (79) | 17.2%  (215) |
|  |  | unknown  % (N) | | 3.9%  (55) | 3.4%  (47) | 2.5%  (35) | 9.8%  (137) |
|  |  | p value** | | - | 0.000 | 0.000 | Goodnes-to-fit=0.000 |
|  |  | OR (95% CI) | | - | 3.1[1.8.-5.3] | 3.3[1.8-5.9] |  |
| Hospitalisation past 6 months | | %(n) | | 56.0% (230) | 51.8%  (208) | 48.6%  (212) | 46.8%  (650)  (unknown n:9) |
|  |  | p value** | | 0.2 | | |  |
| Previous UTI | | %(n) | | 50.2%  (n:265) | 49.5%  (n:217) | 50.5%  (n:213) | 50.1%  (n:695)  (unknown n:5) |
|  |  | p value** | | 0.9 | | |  |
| Antibiotic usage within the past 3 months | | %(n) | | 53.7%  (n:283) | 58.6%  (n:257) | 60.3%  (n:254) | 57.2%  (n:794)  (unknown: 1) |
|  |  | p value** | | 0.7 | | |  |
| Stones | **Bladder** | (+)  %(n)* | | 15.9% (75) | 9.8%  (43) | 6.6%  (25) | 11.6%  (n:143) |
|  |  | unknown  %(n) | | 10.6% (56) | 13.9%  (61) | 10.7%  (45) | (n:162) |
|  |  | p value¬ | | - | 0.06 | 0.000 | Goodnes-to-fit=0.0001 |
|  |  | OR (95% CI) | | - | - | 0.4[0.2-0.6] |  |
|  | **Ureter** | %(n)* | | 14.0%  (66) | 26.5%  (102) | 20.2%  (77) | 19.8%  (245) |
|  |  | %(n) unknown | | 10.8%  (57) | 12.3%  (54) | 9.7%  (41) | 10.9%  (152) |
|  |  | p value¬ | | - | 0.05 | 0.06 | Goodnes-to-fit=0.0000 |
|  |  | OR (95% CI) | | - | 2.1[1.5-2.9] | 1.6 [1.1-2.2] |  |
|  | Calyceal | %(n)* | | 3.6%  (17) | 11.8%  (46) | 5.0%  (19) | 6.6%  (82) |
|  |  | %(n) unknown | | 10.2%  (54) | 11.6%  (51) | 10.2%  (43) | 10.6%  (148) |
|  |  | p value¬ | | - | 0.2 | 0.9 | NS |
|  | **Renal pelvis** | %(n)* | | 13.3%  (63) | 25.3%  (98) | 16.7%  (64) | 18.1%  (225) |
|  |  | %(n) unknown | | 10.4%  (55) | 11.6%  (51) | 9.2%  (39) | 10.2%  (145) |
|  |  | p value¬ | | - | 0.04 | 0.2 | Goodness-to-fit=0.0000 |
|  |  | OR (95% CI) | | - | 1.6 [1.0-2.1] | NS |  |

*omit unknown cases

**Chi-square test

¬Logistic regression analysis by omitting missing values.

# AMR of HAUTIs in Europe (2003-2017)

## AMR Rates of HAUTI Subgroups in Europe – Pooled Sample

A logistic linear regression model was run to determine if there was a difference in the resistance rates of antibiotics per diagnosis. The model was adjusted for the measured confounders (Charlson score, AMR regions (high vs low) and study year.

s-Table 3, AMR rates of HAUTI subgroups and pooled HAUTI sample in Europe.

|  |  | Cystitis  (resistance rate-denominator) | Pyelonephritis | Urosepsis | Overall |
| --- | --- | --- | --- | --- | --- |
| AMX | Pooled sample | 60.4%  (n:397) | 64.2%  (n:321) | 63.8%  (n:296) | 63.4%  (1014) |
|  | *E.coli* | 58.4%  (n:149) | 55.0%  (n:140) | 63.7%  (n:102) | 58.6%  (n:391) |
| AMX+BLI | Pooled sample | 43.8%  (n:331) | 52.1%  (n: 261)  (OR:1.40 [CI:1.01-1.95] p=0.04) | 60.4%  (n: 230)  (OR:1.97 [CI:1.40-2.79] p=0.0001) | 51.1%  (n:822) |
|  | *E.coli* | 45.0%  (n:158) | 41.0%  (n:139) | 58.5%  (n:94) | 46.8%  (n:391) |
| TZP | Pooled sample | 25.0%  (n:252) | 27.4%  (n:201) | 34.6%  (n:179)  (OR:1.62 [CI:1.06-2.47] p=0.03) | 28.5%  (n:632) |
|  | *E.coli* | 21.4%  (n:112) | 19.8%  (n:96) | 35.1%  (n:77) | 24.6%  (n: 285) |
| CXM | Pooled sample | 38.0%  (n:342) | 42.5%  (n:275) | 57.2%  (n: 187)  (OR:2.19 [CI:1.52-3.15] p=0.0002) | 44.0%  (n:804) |
|  | *E.coli* | 22.2%  (n:171) | 28.9%  (n:149) | 41.9%  (n:86)  (OR:2.53 [CI:1.39-4.66] p=0.002) | 28.8%  (n:406) |
| CTX | Pooled sample | 29.2%  (n:318) | 33.8%  (n:275) | 54.1%  (n:194)  (OR:2.90 [CI:2.00-4.22] p=0.0002) | 36.9%  (n:787) |
|  | *E.coli* | 17.7%  (n:158) | 20.6%  (n:150) | 40.7%  (n:86)  (OR:2.49 [CI:1.29-4.86] p=0.006) | 23.9%  (n:394) |
| CAZ | Pooled sample | 27.5%  (n:287) | 27.7%  (n:252) | 44%  (n: 161)  (OR:2.08 [CI:1.39-3.12] p=0.0004) | 31.4%  (n:700) |
|  | *E.coli* | 22.0%  (n:141) | 20.3%  (n:128) | 27.5%  (n:69) | 22.5%  (n:334) |
| CIP | Pooled sample | 41.9%  (n:393) | 44.3%  (n:318) | 58.4%  (n:255)  (OR:1.95 [CI:1.42-2.70] p=0.0004) | 47.1%  (n:966) |
|  | *E.coli* | 39.7%  (n:189) | 37.0%  (n:162) | 59.0%  (n: 100)  (OR:2.12 [CI:1.25-3.64] p=0.0005) | 43.0%  (n:451) |
| LVX | Pooled sample | 40.5%  (n:254) | 37.4%  (n:195) | 59.3%  (n:123)  (OR:2.14 [CI:1.39-3.34] p=0.0006) | 43.5%  (n:572) |
|  | *E.coli* | 37.9%  (n:132) | 27.1%  (n:96) | 57.4%  (n:47) | 37.5%  (n:275) |
| SXT | Pooled sample | 48.1%  (n:339) | 44.2%  (n:267) | 53.9%  (n:228) | 48.4%  (n:834) |
|  | *E.coli* | 44.4%  (n:162) | 37.4%  (n:147) | 53.8%  (n:106) | 44.3%  (n:415) |
| GEN | Pooled sample | 32.4%  (n:379) | 31.6%  (n:319) | 36.9%  (n:265) | 33.4%  (n:963) |
|  | *E.coli* | 21.1%  (n:166) | 18.1%  (n:160) | 22.8%  (n:101) | 20.3%  (n:427) |
| IPM | Pooled sample | 6.7%  (n:282) | 10.0%  (n:238) | 8.2%  (n:195) | 33.4%  (n:715) |
|  | *E.coli* | 5%  (n:118) | 1.8%  (n:111) | 2.3%  (n:88) | 3.2%  (n:317) |
| AMX+GEN | Pooled sample | 23.9%  (n:318) | 22.3%  (n:255) | 28.5%  (n:203) | 24.6%  (n:776) |
|  | *E.coli* | 20%  (n:140) | 15.0%  (n:113) | 19.7%  (n:86) | 18.1%  (n:359) |
| AMX/BLI+GEN | Pooled sample | 21.0%  (n:276) | 21.3%  (n:211) | 30.1%  (n:159) | 23.4%  (n:646) |
|  | *E.coli* | 15.8%  (n:133) | 12.8%  (n:109) | 22.4%  (n:58) | 16%  (n:300) |
| CAZ+CIP | Pooled sample | 21.2%  (n:259) | 21.1%  (n:228) | 41%  (n:144)  (OR:2.79 [CI:1.75-4.47] p=0.000) | 25.7%  (n:631) |
|  | *E.coli* | 16.3%  (n:129) | 16.2%  (n:107) | 29.0%  (n:62).  (OR:2.1 [CI:1.1-4.3] p=0.04) | 18.8%  (n:308) |
| CAZ+GEN | Pooled sample | 17.5%  (n:251) | 15.5%  (n:220) | 29.7%  (n:228)  (OR:2.03 [CI:1.21-3.40] p=0.007) | 19.4%  (n:599) |
|  | *E.coli* | 10.0%  (n:120) | 9.2%  (n:109) | 16.0%  (n:50) | 10%  (n:279) |
| CAZ+SXT | Pooled sample | 17.8%  (n:219) | 21.9%  (n:178) | 32.7%  (n:101)  (OR:2.41 [CI:1.37-4.27] p=0.002) | 22.3%  (n:498) |
|  | *E.coli* | 12.5%  (n:112) | 14.6%  (n:96) | 20.8%  (n:48) | 14.8%  (n:256) |
| TZP+CIP | Pooled sample | 19.3%  (n:228) | 18.8%  (n:181) | 33.1%  (n:145)  (OR:2.08 [CI:1.25-3.45] p=0.004) | 22.7%  (n:554) |
|  | *E.coli* | 15.1%  (n:106) | 13.0%  (n:92) | 32.2%  (n:59)  (OR:2.7 [CI:1.25-5.8] p=0.01) | 18.2%  (n:257) |
| TZP+GEN | Pooled sample | 15.6%  (n:225) | 14.1%  (n:170) | 20.3%  (n:128) | 16.3%  (n:523) |
|  | *E.coli* | 9.2%  (n:98) | 8.9%  (n:79) | 17.4%  (n:46) | 10.7%  (n:223) |
| TZP+SXT | Pooled sample | 15.0%  (n:194) | 16.1%  (n:137) | 22.2%  (n:99) | 17%  (n:430) |
|  | *E.coli* | 8.3%  (n:96) | 9.5%  (n:74) | 19.5%  (n:46) | 11.1%  (n:216) |
| CIP+GEN | Pooled sample | 27.7%  (n:332) | 26.5%  (n:264) | 33.7%  (n:193) | 28.8%  (n:789) |
|  | *E.coli* | 18.9%  (n:153) | 14.2%  (n:134) | 24.6%  (n: 69) | 18.2%  (n:356) |
| CIP+SXT | Pooled sample | 29.1%  (289) | 31.0%  (n:210) | 40.1%  (n:157) | 32.3%  (n:656) |
|  | *E.coli* | 26.1%  (n:148) | 24.1%  (n:116) | 42.3%  (n:71)  (OR:2.05 [CI:1.1-3.7]  p=0.02) | 28.9%  (n:335) |

## Time trends of AMR rates in Europe within the pooled sample of all pathogens

s-Figure 3, AMR rates of single agent antibiotics from pooled sample in Europe per diagnosis.


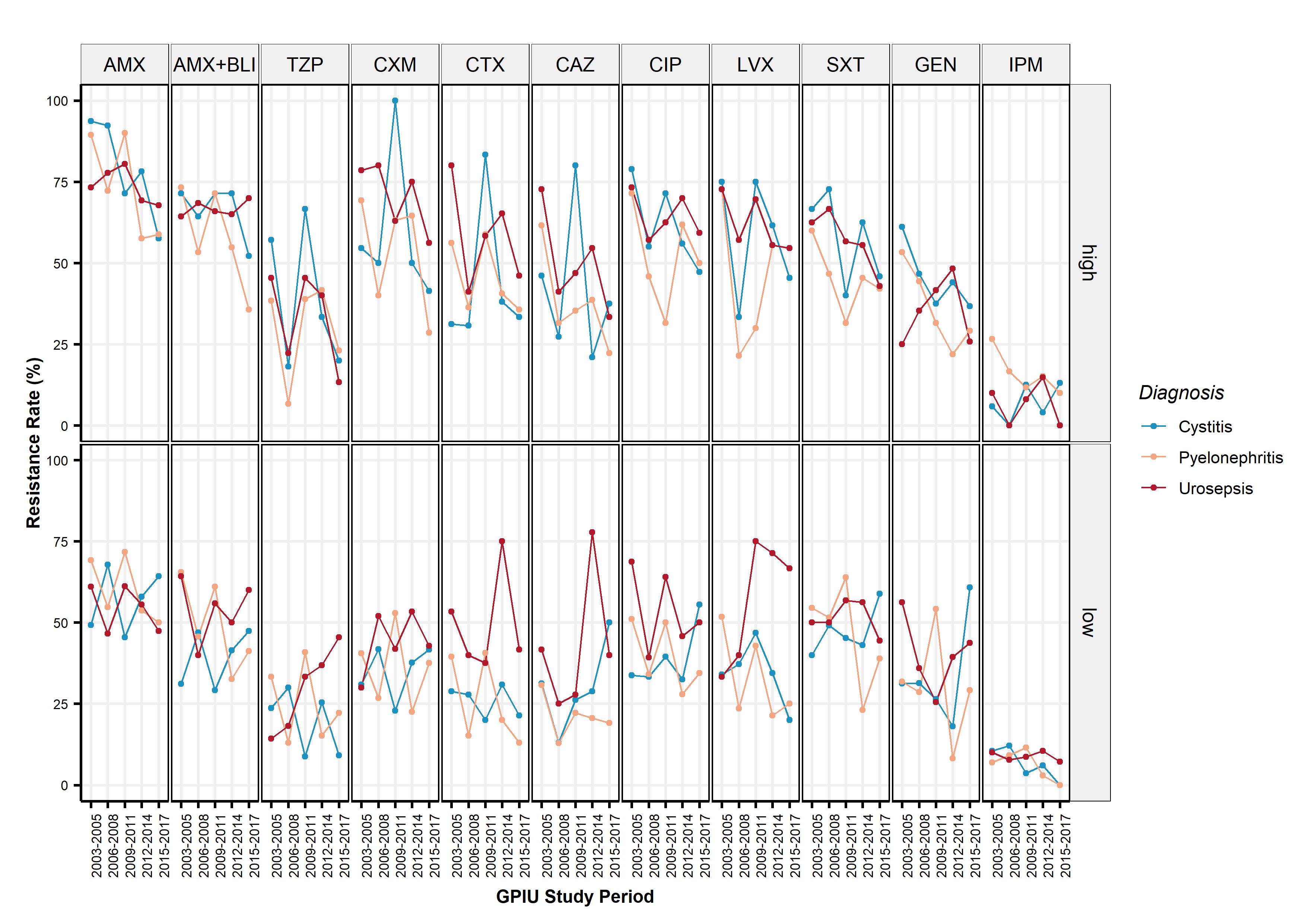


**s-Figure 4, AMR rates of combination agents antibiotics from pooled sample in Europe per diagnosis.**


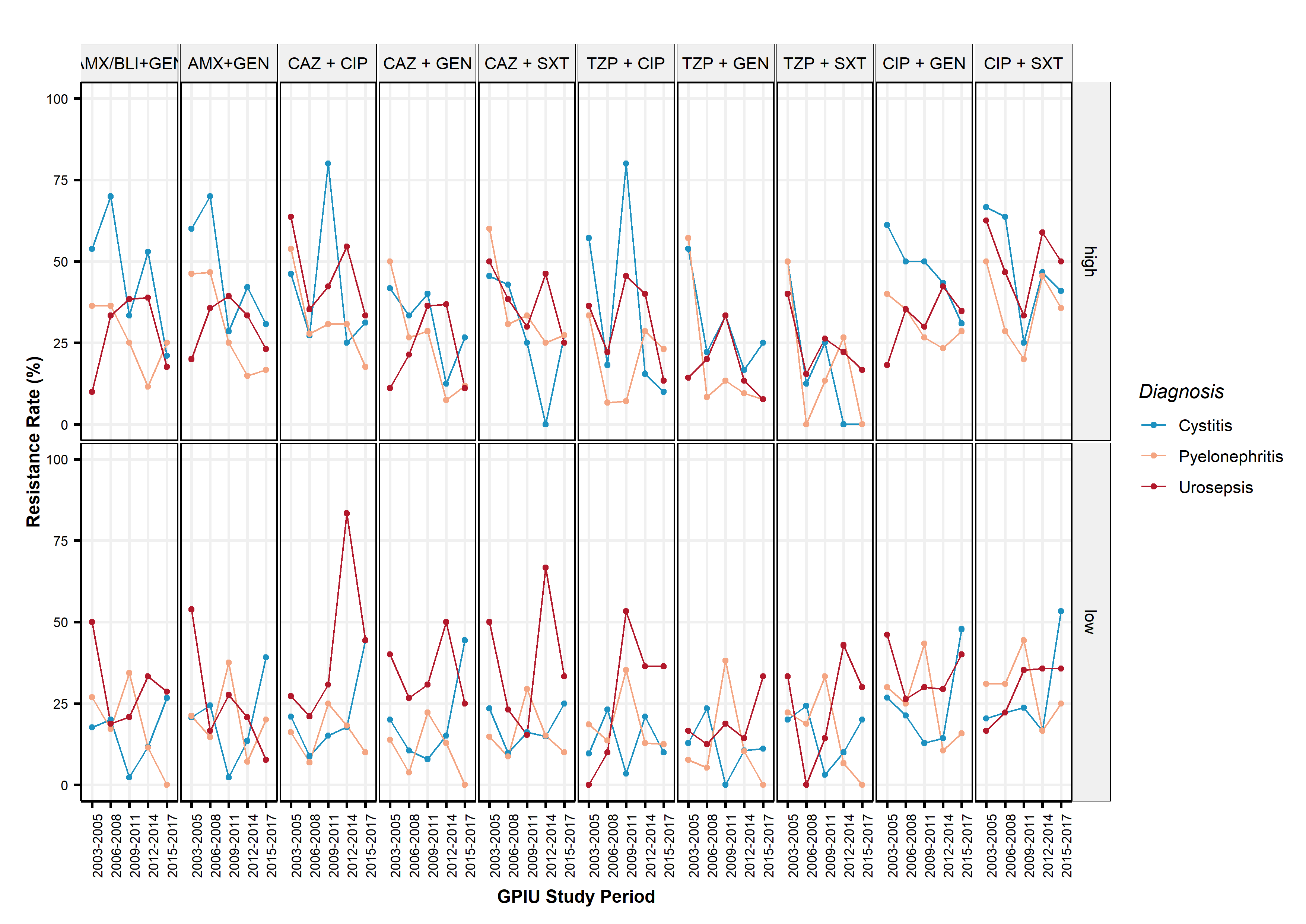


## Time trends of AMR rates in Europe for *E.coli*

s-Figure 5, AMR rates of *E.coli* in Europe per diagnosis.


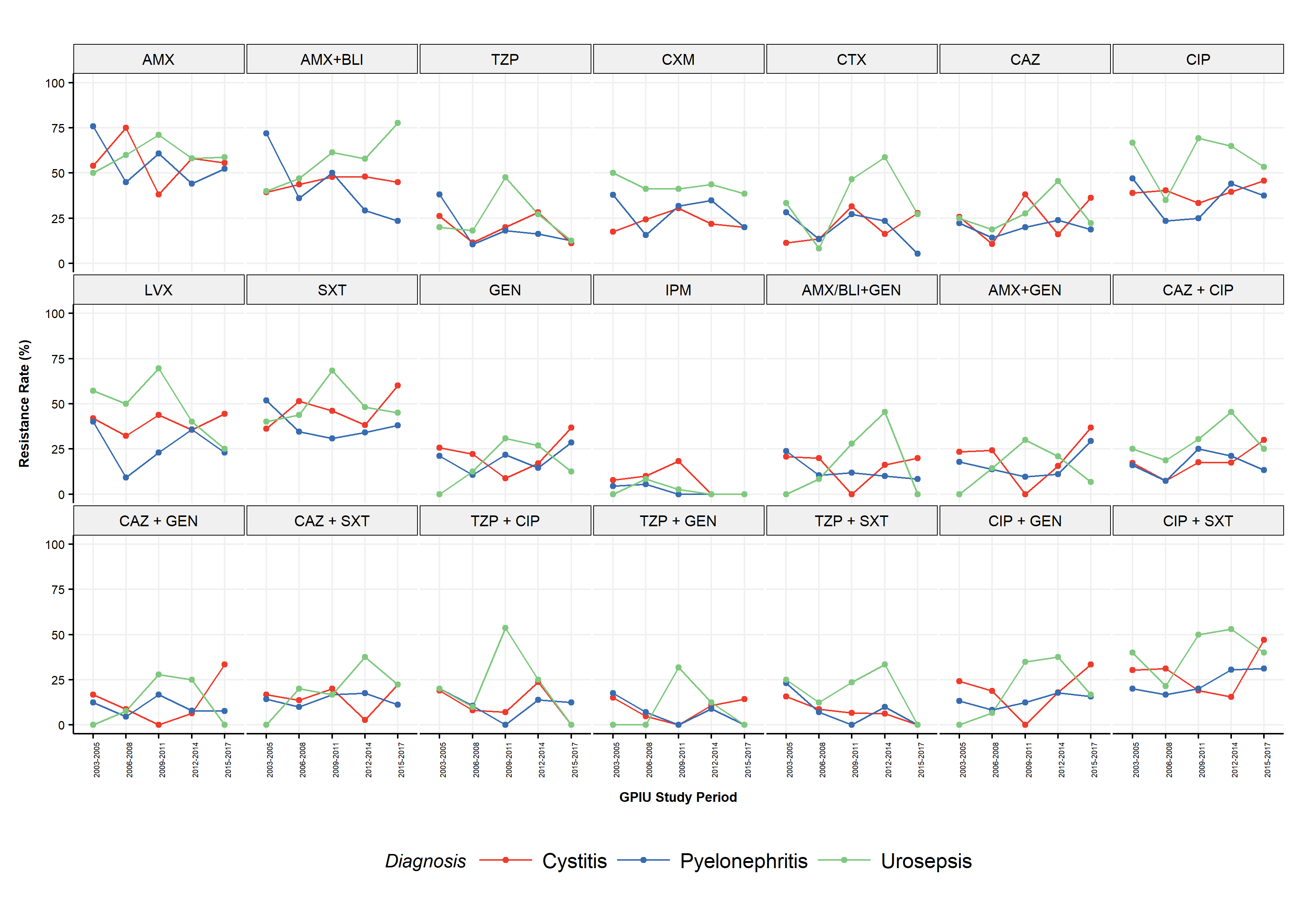


# Charlson Comorbidity Score and Bayesian WISCA Values

s-Figure 6. Annual Bayesian WISCA values of single agent antimicrobial choices for HAUTIs stratified according to Charlson Comorbidity score (<2 vs =>2). These findings highlight two other important findings. First, a lower Bayesian WISCA value in patients with a higher Charlson score. Second, condition specific Bayesian WISCA differences compared to pooled sample values is noted on both categories of patients stratified according to Charlson score.


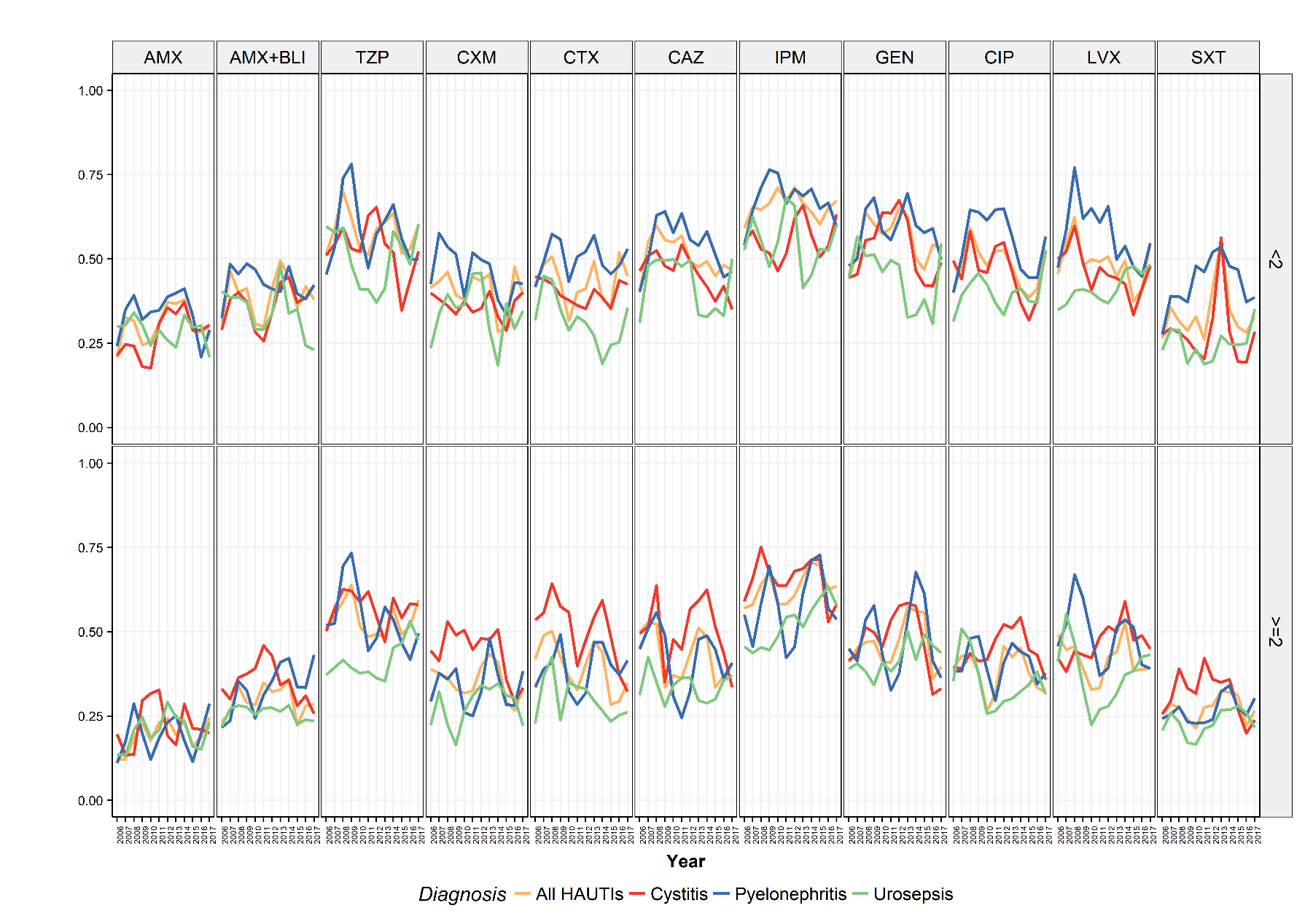


s-Figure 7, Annual Bayesian WISCA values of combination agent antimicrobial choices for HAUTIs stratified according to Charlson Comorbidity score (<2 vs =>2). These findings highlight two important findings. First, a lower Bayesian WISCA value in patients with a higher Charlson score. Second, condition specific Bayesian WISCA differences compared to pooled sample values is noted on both categories of patients stratified according to Charlson score.


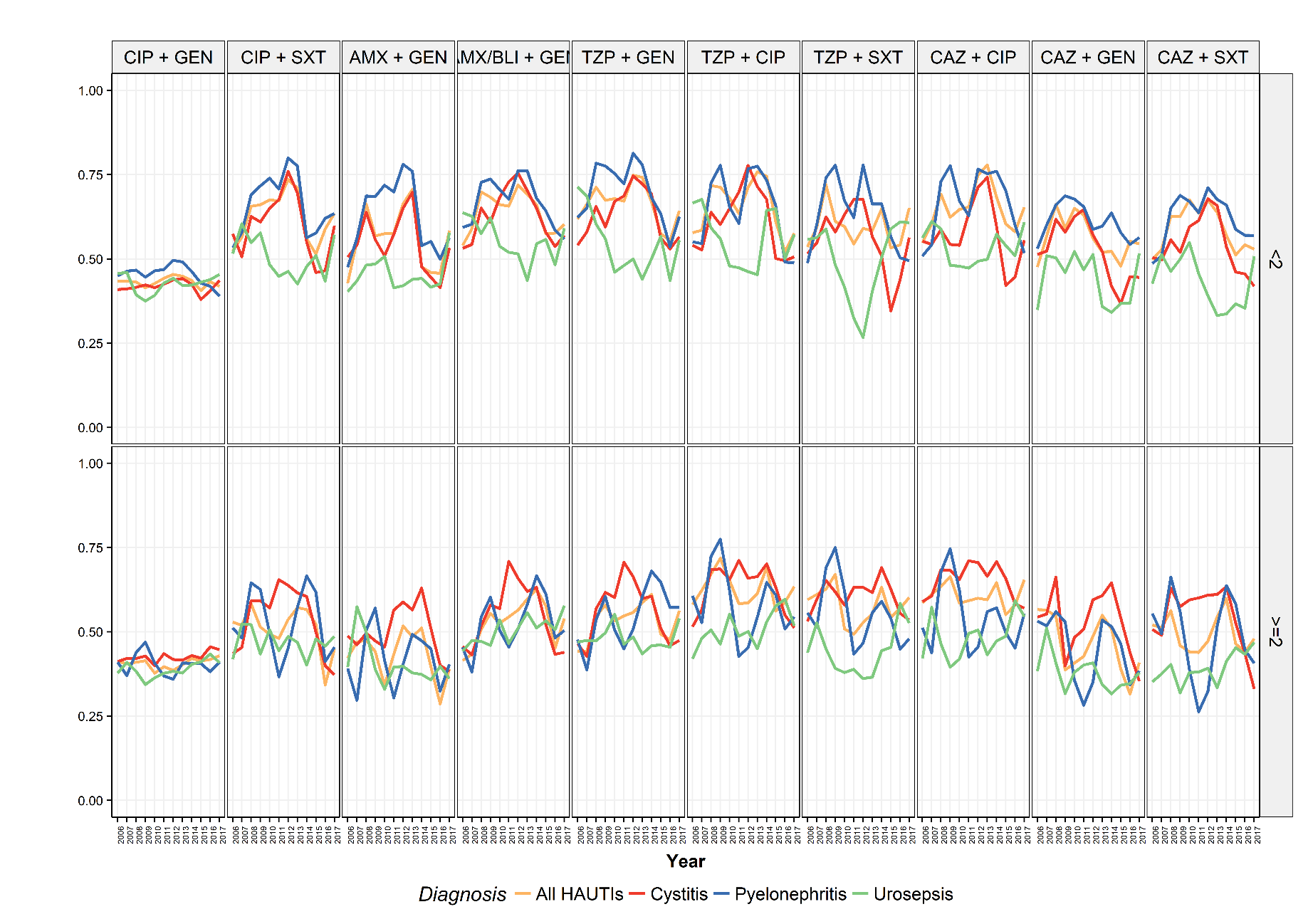

Supplement: Supplementary file 1 — Supplementary material 1 (DOCX 2385 kb) [file 345_2019_2963_MOESM1_ESM.docx]
